# Report of Illinois State Board Meeting

**Published:** 1884-08

**Authors:** 


### SELECTIONS.

The regular quarterly meeting of the Illinois State Board of Health was held in its rooms in the Capitol Building, at Springfield, on Wednesday, July 2, 1884.

Present, at the afternoon session, Drs. Haskell, McKensie, Kreider and Rauch; Dr. Haskell presiding in the absence of the president; and at the evening session, in addition to the above, Newton Bateman, the president, in the chair.

Dr. Geo. N. Kreider, of Springfield, appointed to succeed Mr. Reen, of Peoria, resigned, was introduced to the members and took his seat with the Board.

The minutes of the last quarterly meeting, April 17 and 18, were read and approved; after which the regular order of business was suspended and the Board went into executive session on certain cases of colleges and practitioners, under the Medical Practice Act.

During the evening session the following quarterly report of the Secretary was presented:

QUARTERLY REPORT OF THE SECRETARY, APRIL 1–JUNE 30, 1884.—During the quarter ending June 30, 1884, there were received in the Secretary's office 604 communications, letters, papers, etc., exclusive of 197 diplomas submitted for verification, and the affidavits and other papers accompanying applications for certificates in 226 cases. There were sent out during the same period, 827 letters, postals, circulars, etc., and other communications, and the usual quantity of the Board's publications—reports, registers, preventable-disease circulars, epidemic disease blanks, forms of ordinances, etc. Two hundred and forty-two packages were received, and 212 sent out, by express. Seventy-three telegrams received and 102 sent.

Certificates entitling to practice medicine and surgery, under the Medical Practice Act, were issued to 170 graduates upon diplomas of colleges which have complied with the requirements of the Board, entitling them to be classed as in good standing, and to four upon length of practice in the State. Under the authority conferred upon the Secretary at the last meeting, seventeen applicants for certificates, holding diplomas of colleges which had not fully complied with the Board's requirements, have been notified that they would have to submit to examinations on the branches or subjects omitted by their respective schools. In nine of these cases the applicants have already been examined and certificates issued; five of these were examined in hygiene only; three in hygiene and general education; and one on all the branches, including general education. Three declined to appear to be examined and have left the State, and the remaining five are now awaiting their examination.

Examinations of five midwives have been made, and certificates issued to three of these, who passed successfully, and seven to others upon diplomas and licenses, or other recognized credentials.

THE MEDICAL PRACTICE ACT.—Since the last meeting of the Board, the Dr. C. Buell Rice, to whom a certificate was refused at the special meeting of January 30, has been tried and convicted of practicing in violation of the Medical Practice Act. The case was tried in the Sangamon County Court, May 19, before his Honor, Judge Matheny.

The defense set up the plea that, being a graduate of a "legally chartered medical institution in good standing," the defendant was entitled to a certificate; and that it was not competent for the Board to enquire into the moral or professional character of such graduates. On the part of the prosecution, it was shown that charges had been presented to the Board, alleging that Rice was in the employ of, and associated with, the "K. and K. Surgeons," a firm of advertising quacks, of Cincinnati and elsewhere, and that, in various ways connected therewith, as recited at the special meeting, January 30, his conduct was unprofessional and dishonorable, within the meaning and intent of the Medical Practice Act; that upon these charges the Board had refused to issue Rice a certificate until he had disproved the same; that instead of making any attempt at such disproof, Rice continued to practice; whereupon he was arrested for practicing without the necessary certificate. The facts were admitted by the defense, but, as already stated, the Court was asked to dismiss the suit on the ground that it was obligatory on the Board to issue its certificate to the possessor of a genuine diploma of any "legally chartered medical institution in good standing," regardless of the moral or professional status of the individual. This the Court declined to do, but found the defendant guilty, and assessed a penalty of $50.00 and costs. Notice of appeal was at once given by the attorneys for the defense, but this was subsequently abandoned, the fine and costs were paid, and this last representative of the "K. and K. Surgeons" has left the State.

While this decision again affirms the right and duty of the State Board of Health to inquire into and determine the status of individual practitioners, a decision by the Supreme Court of the State, rendered May 20, sustains the right of Boards constituted as is the State Board of Health to determine the status of a college. Under the Act to Regulate the Practice of Dentistry in Illinois, the Supreme Court refused the petition of a dentist, one Isaac N. Sheppard, for a writ of mandamus to compel the State Board of Dental Examiners to issue him a certificate or license based upon a diploma of the Indiana Dental College. The Board refused the license upon the ground that the College was not a "reputable" institution. It was argued that the law constitutes the Board judges of the standing of a college, and there is no power of review vested in any other body.

"If the Board should arbitrarily or unreasonably abuse their discretion, and refuse a license without any reason therefor, there is a remedy for such abuse of discretionary power." But there was no ground for claiming that this was the case in the present instance. The Board, in its judgment, had decided that the curriculum of study and requirements for graduation of the Indiana Dental College were not such as to entitle it to be classed as "a reputable dental college," and there is no power in the law given to any person or body to review and set aside, or confirm, the exercise of this discretion by the Board. The petition for a mandamus was, therefore, denied.

It is also noted, in this connection, that two colleges which the Board has long refused to recognize as in good standing, have recently met with signal and final defeat in their efforts to secure a legal rehabilitation. The New York Court of Appeals has affirmed the judgment rendered by the Supreme Court of that State, about a year ago, in the case of the United States Medical College of New York, setting aside the charter of that institution. As this appeal was understood to be taken as a test case by the attorneys for the Buffalo College of Physicians and Surgeons, this decision is to be regarded as conclusive on this college also.

There have been fewer complaints, made direct to the Board, of unprofessional conduct, and fewer cases of this kind otherwise coming under observation, during this quarter than ever before in the history of the Board. The man J. E. Anderson, of the "American Surgical Institute," of Indianapolis, previously run out of Paxton, Tolono, and elsewhere, was arrested at Freeport, on the 9th of May, for practicing in violation of the law. The case was clearly made out, Anderson pleaded guilty, a fine of $50 and costs was assessed, which he paid, and at once departed for his Indiana home. A list of some fifty of his victims in the northern part of the State, has been furnished me. About the middle of June an itinerant, by the name of Riley, was arrested in Dixon for violation of the law, and was bound over for trial.

Two of the Chicago quacks, arrested for circulating obscene and indecent literature through the mails, have recently been tried in the United States District Court, and fined $200 each, with costs. One of these cases was an aggravated one, many of the vile pamphlets having been sent to school-girls at various places. I do not hesitate to pronounce the penalties inflicted in these cases as totally inadequate. Although the stereotype plates and the editions found were understood to have been destroyed, one of these men is already distributing his "Hidden Secrets." The only way to suppress these violators of public decency and morals is to imprison them.

MEDICAL EDUCATION.—At the annual meetings of the various medical organizations, State and National, which have been held during the past three months, the subject of the preliminary education of medical students has received more than usual attention. Almost unanimously, the individual members of the profession, and these various organizations as units (with one single exception), have pronounced in favor of exacting proof of proper preliminary education before admitting candidates to the lecture classes. There is practically no opposition to the movement, the only dissentients now remaining being a few members of college faculties, influenced, probably, by a fear of diminishing classes. With few exceptions—and these diminishing in number from time to time—the better class of colleges have already adopted this requirement. Every announcement for the session of 1884–85, thus far received, makes this a distinctive feature; but it is to be wished that the colleges would state specifically in their announcements the kind of examinations applicants would be subjected to, or the proof of fitting education required, instead of merely saying, as some of them do, "a preliminary education is required," or making use of some similar vague generality.

As illustrating the wide-spread influence of the effort to heighten the standard of professional acquirements, it may be stated that, at a recent meeting of the Nebraska State Medical Society, the qualifications for admission to membership were so amended as to require that applicants must be graduates of colleges which in all respects conform to the standard of minimum requirements of this Board.

In the further interest of medical education, I think it the duty of the Board to exert its influence towards securing legislation for the proper and adequate supply of material for the study of practical anatomy. Colleges in this State have been embarrassed, during the past year or two, in their efforts to properly instruct their students in this most important branch; and the difficulty is increasing. Surgical knowledge and skill cannot be acquired without an intelligent practical study of anatomy; and in order to secure this, the methods and sources of the supply of material need to be more definitely recognized and regulated by law.

THE PUBLIC HEALTH.—Scarlet fever and small-pox prevailed to some extent during the first half of the quarter, mainly in the southern portion of the State. Except the few cases in Chicago and those in Kendall county, all the small-pox cases occurred in the south half of the State, but scarlet fever was more generally diffused. Both diseases have been of a mild type, with a moderate death rate. As the season advanced there has been the usual increase in the diseases of hot weather, but not characterized by any noteworthy features.

Although eight cases have been brought into Chicago from other places since January 1st, 1884, only one case was contracted from any of these by a resident of the city. Three of these eight cases were Indians brought in from the Indian Reservation; two were from Indianapolis; one from Cincinnati; and two from the town of Cicero—said to have been contracted from a tramp from Ohio. One of the Indianapolis cases reached Chicago four days before the appearance of the disease, and from him resulted the only case which originated in the city—a man with whom he slept one night contracting the disease from him. In no other case was there any spread of the disease; and the methods of dealing with cases as they appear, the details of disinfection, the general vaccinal protection of the community—especially of the 70,000 school children and the large number of poor people—are so thorough that Chicago, notwithstanding its immense railroad travel and large number of transients, has been, for many months, one of the safest cities in the Union in this respect.

At the close of the last quarter small-pox existed in Centralia, Marion county; Charleston, Coles county; and Coulterville, Randolph county. Owing to municipal neglect and a mistaken idea of economy, the disease obtained a foothold in Centralia, which it subsequently required great effort to overcome, besides creating alarm and apprehension in neighboring communities. The disease was conveyed from this place to Irvington township, in Washington county; to Bellerive, in Jefferson county; and to Springfield. The first case in Centralia, it is stated, was treated by Dr. Alexander Jones, whose certificate the Board revoked at the January meeting, for unprofessional and dishonorable conduct. It is alleged that Jones treated the case without any of the necessary precautions; not reporting it to the authorities, nor in any manner guarding against spread of the contagion. The patient was treated during the entire illness in a room separated only by a curtain from a shoe shop on one of the most frequented streets of the town, and the shop was daily visited by numbers of persons. It is believed that all the cases in Centralia—32 in number, with 6 deaths—as well as those in Washington, Jefferson and Sangamon counties, are primarily due to the criminal conduct of this man. The Board has exhausted its authority in dealing with him by revoking his certificate, since he claims the exemption of the ten years prior practice clause of the Medical Practice Act. In charging him, however, with being primarily responsible, it is not meant to exonerate the municipal authorities from all blame; for, as early as the 8th of March, they were duly notified of the existence of a case resulting from this first concealed case.

From the tramp who carried the disease into Charleston, Coles county, as reported at the April meeting, there resulted five other cases, making a total of six cases and three deaths. The first of this last group of cases was a man who visited the tramp, denying that the latter had small-pox; in the usual time he came down with the disease, and died on the thirteenth day.

At Coulterville, Randolph county, also mentioned in my last report, there were five cases, with one death in the first outbreak, which was caused by a negro roustabout who had contracted the disease on the river. Notwithstanding a rigorous quarantine of isolation and other precautionary measures, some obscure cases of varioloid followed this first outbreak; and, through failure to correctly diagnose the early cases of this second group, which were not characteristic, the disease still continues. The condition of affairs at this place, and a conflict of opinion as to some of the cases now under treatment, led me to visit the locality personally on the 28th of June, when I found two well-marked cases, one of small-pox and one of varioloid. The severer of these two—and which will probably prove fatal—was in Perry county, just over the line; but instructions were given the Coulterville authorities to extend their quarantine jurisdiction so as to embrace this case, and to vaccinate or revaccinate all persons in the compromised area, who had not been successfully protected within the last two and a half years. The spirit manifested by the village authorities, the physicians and the citizens whom I met, warrant the belief that this outbreak will now be soon suppressed. I communicated also with one of the county commissioners and the county clerk of Perry county, and the county commissioners of Randolph county, and feel assured of their co-operation and assistance.

The outbreak at Yorkville, Kendall county, previously alluded to as having been first reported at the close of the last quarter, was due to a young man recently arrived from New Orleans, who had an unrecognized case of varioloid. A large number of persons were exposed before the facts were known, and a total of nineteen cases, with four deaths, resulted. So much excitement was caused by the first group of these, some fourteen in number, eleven of which appeared in rapid succescession between March 27th and April 2d, and in several localities, that I found it necessary to visit the town personally. The published instructions of the Board were thoroughly carried out, a supply of vaccine virus was obtained, and all unprotected persons were at once vaccinated. Notwithstanding the number of the centers of infection, only five more cases resulted—the last being after an interval of fully a month. The speedy suppression of the outbreak, which promised to be very serious at the date of my visit, was due to the prompt and efficient co-operative action of the authorities of the four separate jurisdictions in which the cases appeared. The detailed reports of these cases have been received, and from them the usual state of facts concerning vaccination is found; that is, that of the four fatal cases, three had never been vaccinated at all, and the remaining one had had small-pox when young, and had been vaccinated "seven or eight times unsuccessfully—it never would work"—owing probably to the previous attack of small-pox, the protection from which seems to have been exhausted prior to this last exposure. Of the fifteen who recovered, two had never been vaccinated at all; one, not until after the febrile stage of the disease had begun; and four others, not until after exposure. All these recovered, as did also the remaining eight who had been successfully vaccinated at various periods prior to exposure. None of those attacked had ever been revaccinated.

There have been seven cases of small-pox, with two deaths, among colored steamboat hands at East St. Louis, all contracted on the river. These cases were removed as soon as discovered, from time to time, to the St. Louis small-pox hospital; and about the last of May I received a communication from the Health Commissioner of St. Louis, stating that the East St. Louis patients were the only ones then in the hospital, and that the institution was kept open solely for the benefit of the latter place. While this is technically true, the relations of the two places are such that what is done for the one, in such a matter as this, is really done for both. However, I again visited East St. Louis and discussed the situation with the authorities, and am glad to be able to state that a Board of Health has since been organized, and the burial-permit ordinance prepared by the Board has been adopted.

Isolated cases of the disease have also occurred during the quarter at Mound City, Pulaski county—one case, probably contracted on the river, no spread; Red Bud, Randolph county—one case, contracted in Cairo, no spread; in Irvington township, Washington county—two cases, contracted in Centralia, and one from these in the person of the nurse, who had had the small-pox when young; at Bellerive, Jefferson county—one case, contracted in Centralia, no spread; in the country, six miles north of Nashville, Washington county—one case, contracted in Coulterville, and from this four more cases in the same family; in Ashmore township, Coles county—one case, contracted in nursing an unreported case on the county poor-farm, patient had had small-pox when young; and in Springfield, Sangamon county—one case, contracted in Centralia, and not recognized until after death, so that there will probably be other cases from this, although the usual precautions were at once taken, when the character of the illness was discovered. At the close of June another group of three cases is reported in East Fork township, Clinton county, five miles southwest of Patoka, and sixteen miles north of Centralia, where the disease was contracted.

Although the contagion has been repeatedly introduced into Illinois from without, during the past winter and spring, we have been fortunate in escaping it by immigrant introduction, thus far. Neighboring States have been less favored—Iowa, for example, having now a serious outbreak, upwards of 20 cases in one county—all among emigrants landed at Baltimore, from the steamer *Salier*, of the North-German Lloyd's Line. The want of appropriations, whereby the National Board of Health might continue its Immigrant Inspection Service, is seriously regretted.

With regard to further action concerning small-pox, in view of the probability of its epidemic spread from abroad, as shown by its increasing prevalence in London and elsewhere, and its frequent introduction into the State from neighboring States, I would suggest that it is desirable to call the attention of sanitary authorities and others to these facts and to the experience of the last few months, which shows that when the disease is introduced into a community where vaccination and revaccination were not thoroughly carried out during the recent epidemic, there is still danger of serious trouble. It is also important that County Superintendents, school-boards and others interested should have their attention again directed to the fact that the School Vaccination Order of the Board is permanent and continuous; and that its thorough enforcement is expected, so as to prevent any accumulation of unprotected or imperfectly protected scholars from term to term. To this end I think it necessary to again print and distribute copies of the Order, with necessary instructions, together with supplies of certificates and blanks for returns to be made through the county superintendents by the first of January next.

Cases of glanders and other infectious diseases among animals continue to be reported to the Board. On the 28th of June Dr. C. N. Cooper, of Batavia, Kane county, reports having a patient under treatment, suffering with glanders, and wishes instructions and advice as to his actions. The amended Pleuro-pneumonia and Glanders Act has by no means relieved the Board of responsibility in these cases.

The public naturally apply to the health authorities in matters pertaining to health, and thus far the State Veterinarian is only reached in a large number of cases through this office. Whether legislation is necessary on this subject, and what form it should take, are matters which seem to require the consideration of the members.

CHOLERA.—An epidemic spread of Asiatic cholera now seems imminent. What is known as the Damietta outbreak failed to secure a foothold in Europe last year, and with the exception of a few isolated cases in Russia and one fatal case at the Smyrna lazaretta—all in July of 1883—it is believed that the disease from this outbreak was confined to the delta of the Nile. France, it is true, was threatened by the arrival at Havre of the steamer *St. Bernard*, in June last, with one case on board; but preventive measures were successfully instituted on that occasion and the evil then averted. About the first of May, 1883, the British troop-ship *Crocodile* was reported in quarantine at Portsmouth, England, having then had eight cases of cholera on board, six of which proved fatal; but on this occasion, also, the disease seems to have been confined to the infected vessel. The French have been less successful in their recent precautionary attempts, if it be true, as is now alleged, that the Toulon outbreak was due to a fatal case on board the transport *Montebello* from China—the infected clothing of the case not being destroyed. Later advices state that the disease was brought from Egypt in the troop-ship *Sarthe*. It has already spread to Marseilles, the villages above Toulon, and cases are reported in Italy and elsewhere.

Whether the disease will cross the Atlantic from the east will largely depend, of course, upon the efficiency of the measures employed to confine the contagion to its present localities. Very general activity is manifested by all the European governments and sanitary authorities; and it is to be hoped that they may prove successful, although the dissenting opinions of the English authorities as to quarantine may lead to friction between them and the Continental authorities. Meanwhile we are threatened not only from Europe, but from the opposite side of the globe, cholera seeming to be spreading in China, and to have broken out in Japan.

In view of this condition of affairs, I have thought it my duty, on behalf of the Board, to urge that the organization of the National Board of Health should be maintained, and have advised to that effect, hoping that it may be possible to still secure the necessary appropriation for this purpose in the Sundry Civil Service bill. Should cholera continue to spread on the Continent, it is more than likely to find an entrance into this country, despite the efforts which may be made by local and State authorities to exclude it by quarantine regulations. Want of uniformity, failure to co-operate, commercial considerations, and local conditions, all combine to impair the efficiency of any system of quarantine; and to the extent that these obtain, in the absence of a uniform system, is the danger of failure increased. The remedy, of course, is in the National control and administration of quarantine; and the present emergency furnishes another argument for the continuance of the National Board of Health, with adequate appropriations and increased power and authority.

As to what should be done by us as a Board in the present aspect of affairs, I would say that my own experience and observation lead to the conclusion that it is not judicious to place entire reliance on quarantine measures, no matter how administered, should the disease become epidemic in countries or ports with which this country has close commercial relations. Asiatic cholera, although it may invade places in good sanitary condition, finds its most congenial habitat where filth in any form abounds. The best attainable sanitary condition—clean streets and premises; the prompt and proper disposal of organic refuse, night-soil and all forms of sewage; well ventilated habitations with dry, clean basements; a pure and sufficient water-supply; and good individual hygiene, including personal cleanliness, a proper diet, and regular habits of life—these are the best safeguards against Asiatic cholera, as they are against most diseases. If it should unfortunately appear in a locality whose sanitary condition is good, as thus outlined, there is every reason to anticipate its prompt arrest by well understood measures—thorough isolation of cases, disinfection of discharges, etc. Cholera is pre-eminently a disease to be fought by sanitation.

Professor Koch's recent researches, by which he has demonstrated the existence of the cholera bacillus, promise something in the way of special prophylaxis; and in so far as this is in a direction to which a great mass of empirical knowledge points, it may be worth while calling attention to this feature. I allude to the practical point, which Koch has demonstrated, of the development of the coma-bacilli in alkaline moisture, and its arrest or destruction by acids. If further experiments confirm this proposition, the preventive treatment of Asiatic cholera may come to be a matter of as much certainty as the prevention of small-pox.

As to our own immediate action in the premises, I have to suggest that the attention of sanitary authorities throughout the State be officially called by the Board to the necessity of promptly securing the best attainable sanitary condition of their respective localities.

A thorough sanitary organization of the State, with the view of improving its general sanitary condition, is one of the matters to which the Board may now profitably give its attention. The Board is now, at the end of its first term, in a position to give this subject more consideration than heretofore; and it is entirely possible that such an emergency may present itself as will make this of the first importance.

YELLOW FEVER.—With the exception of recent reports of the reappearance of the disease at Guaymas, Mexico, where it prevailed extensively last year, and of some increase in the number of cases in Havana, there is an encouraging absence of yellow fever indications in the South up to date. A conference of representatives of the Boards of Health of the Gulf States was held, at the request of the newly organized Louisiana State Board of Health, in New Orleans, on the 2nd, 3rd and 4th of June; the object of the conference being to bring said boards into harmony, and, if possible, to devise and recommend improvements in the systems of quarantine in vogue along the Gulf coast. Representatives of the General Government, of the Auxiliary Sanitary Association, and of the various commercial organizations of New Orleans, were also present by invitation. The proceedings were characterized by moderation and a gratifying display of mutual confidence and desire for thorough co-operation. Many practical suggestions were made, and, on the whole, the conference would seem to promise more of benefit than anything of the kind which has occurred of late years in that region—provided, the means necessary to put into effect the wishes and intentions of those concerned be forthcoming.

Meanwhile, as already stated at our last meeting, the Sanitary Council is prepared to adopt, if it should become necessary, the same line of action in regard to the prevention of the introduction of yellow fever, or other epidemic diseases, into the Mississippi Valley, as that pursued in 1883.

SANITARY CONFERENCE.—In accordance with the authority given at the last meeting, I attended the conference of representatives of State Boards of Health, held during the recent session of the American Medical Association in Washington. An organization was effected, officers elected, and plans adopted for securing co-operative action by the various Boards in the event of any emergency arising which threatens the sanitary interests of any of the States in common. Meetings will be held during the annual sessions of the American Public Health Association for the interchange of views and the furthering of the plans and objects of the organization.

THE FIFTH ANNUAL REPORT.—Continued delays in the public printing office have still further postponed the publication of the Fifth Annual Report of the Board; but a full force of compositors is now at work upon it, and assurance is given of its speedy completion.

The material for the Sixth Annual Report is in an advanced state of preparation, and together with the Revised Official Register of Physicians and Midwives, which is also nearly ready, will be put in the printer's hands as soon as the Fifth Annual Report is out of the way.

All of which is respectfully submitted.

JOHN H. RAUCH,
Secretary.

At the conclusion of the reading of the Secretary's report, which was accepted, and ordered placed on file, the following resolutions were adopted, looking to putting into effect the various suggestions embodied in the report.

*Resolved*, That the importance of the study of practical anatomy, as a foundation for surgical knowledge and skill, demands that the supply of material for this study be more definitely regulated, and its necessity recognized by law, and that the Illinois State Board of Health respectfully urges the attention of law makers to these considerations.

*Resolved*, That the increasing prevalence of small-pox in London and elsewhere, indicating a probable renewal of this epidemic tendency, and its frequent introduction into Illinois from neighboring States, within the past few months, make it desirable that vaccinal protection be secured as fully as possible in every portion of the State; and to this end the Secretary is hereby authorized to call the attention of the sanitary authorities and others to the subject; and to take the necessary steps to push the further enforcement of the School-Vaccination Order of the Board, so that all new scholars, and those who have not heretofore fully complied with its provisions, may be properly protected against small-pox before the advent of cold weather.

*Resolved*, That while epidemic cholera may be excluded from this country by thoroughly-enforced quarantine regulations, yet the best attainable sanitary condition of every locality in the State should be secured, so that, in the event of Asiatic cholera effecting an entrance, notwithstanding quarantine, the disease may be met and fought under the most favorable circumstances. The Secretary is, therefore, hereby authorized to take such action as, in his judgment, will most promptly obtain a thorough sanitary organization of the State and the adoption and enforcement of the measures necessary to improve its general sanitary condition.

On motion of Mr. Haskell, the Secretary was given discretionary authority to act for the Board in any case of emergency which may arise in the interims between the regular meetings.

During the executive sessions the cases of a number of colleges, with reference to the requirements of the Board and their standing under the Medical Practice Act; the important feature of the office correspondences during the quarter; and the cases of a number of practitioners, were considered, and the necessary action was taken in a large number of these.

The following certificates were ordered to be revoked:

Certificate No. 913, issued to Peleg W. Blakeley.

Certificate No. 1103, issued to Fritz Tripple.

Certificate issued to William Becker.

Certificate No. 3190, issued to S. Meyer.

After the transaction of sundry routine business, auditing of accounts, etc., which occupied the hours of the morning session, the Board ajourned at 11:30 A. M.

